# Ribosomopathies: New Therapeutic Perspectives

**DOI:** 10.3390/cells9092080

**Published:** 2020-09-11

**Authors:** Emilien Orgebin, François Lamoureux, Bertrand Isidor, Céline Charrier, Benjamin Ory, Frédéric Lézot, Marc Baud’huin

**Affiliations:** 1 INSERM, Bone sarcomas and remodeling of calcified tissues, Nantes Université, UMR 1238, F-44000 Nantes, France; emilien.orgebin@univ-nantes.fr (E.O.); francois.lamoureux@univ-nantes.fr (F.L.); celine.charrier@univ-nantes.fr (C.C.); Benjamin.Ory@univ-nantes.fr (B.O.); frederic.lezot@univ-nantes.fr (F.L.); 2 CHU Nantes, Service de Génétique Médicale, F-44000 Nantes, France; Bertrand.ISIDOR@chu-nantes.fr

**Keywords:** ribosomopathies, riboprotein, treatment, ribosome

## Abstract

Ribosomopathies are a group of rare diseases in which genetic mutations cause defects in either ribosome biogenesis or function, given specific phenotypes. Ribosomal proteins, and multiple other factors that are necessary for ribosome biogenesis (rRNA processing, assembly of subunits, export to cytoplasm), can be affected in ribosomopathies. Despite the need for ribosomes in all cell types, these diseases result mainly in tissue-specific impairments. Depending on the type of ribosomopathy and its pathogenicity, there are many potential therapeutic targets. The present manuscript will review our knowledge of ribosomopathies, discuss current treatments, and introduce the new therapeutic perspectives based on recent research. Diamond–Blackfan anemia, currently treated with blood transfusion prior to steroids, could be managed with a range of new compounds, acting mainly on anemia, such as L-leucine. Treacher Collins syndrome could be managed by various treatments, but it has recently been shown that proteasomal inhibition by MG132 or Bortezomib may improve cranial skeleton malformations. Developmental defects resulting from ribosomopathies could be also treated pharmacologically after birth. It might thus be possible to treat certain ribosomopathies without using multiple treatments such as surgery and transplants. Ribosomopathies remain an open field in the search for new therapeutic approaches based on our recent understanding of the role of ribosomes and progress in gene therapy for curing genetic disorders.

## 1. Introduction

Ribosomes, complexes composed of ~80 proteins and four rRNAs, forming two distinct subunits, are key actors in the development of cells’ proteome [1]. Their role is not limited to the translation of mRNAs, but also to folding the newly-formed polypeptidic chains and ensuring the synthesis of correctly shaped proteins (as reviewed by [2]).

**Figure 1:**
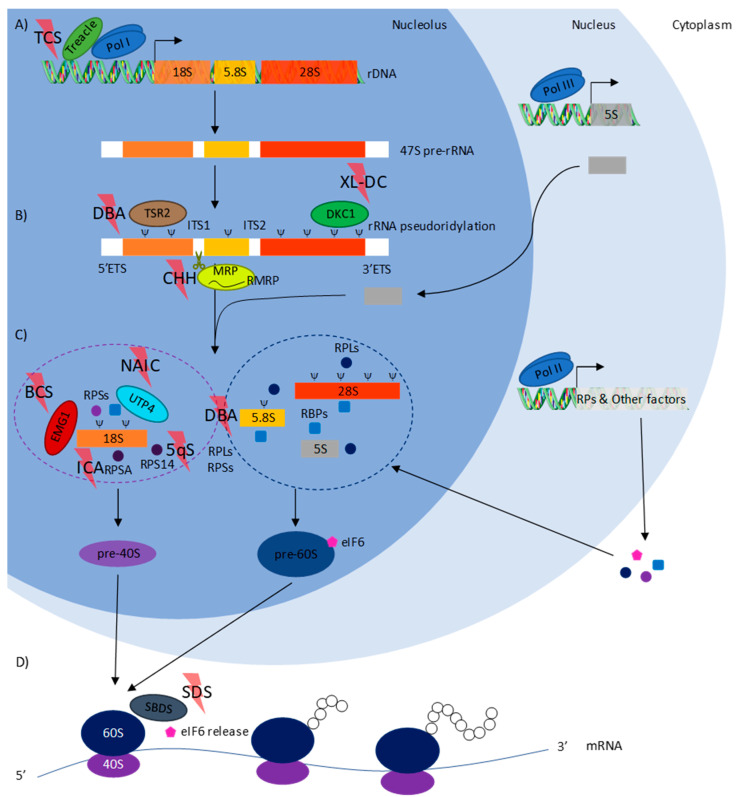
Eukaryotic ribosome biogenesis pathway. (**A**) In the nucleolus, rDNA is transcribed into 47S pre-RNA by RNA polymerase I associated with Treacle protein, which is mutated in Treacher Collins syndrome (TCS); (**B**) 47 pre-RNA is then pseudouridinylated by a complex containing DKC1, which is mutated in X-linked dyskeratosis congenita (XL-DC). Complete maturation of rRNAs requires the removal of external (ETS) and internal (ITS) transcribed spacers. RNase MRP, containing RMRP, which is mutated in cartilage hair hypoplasia (CHH), cuts in site 2 (ITS1) transforming 45S pre-rRNA into 30S and 43S pre-rRNAs, but also 41S pre-rRNA into 21S and 32.5S pre-rRNAs through two separate pathways. TSR2, mutated in Diamond–Blackfan anemia (DBA), may be responsible for the processing of 18S rRNA; (**C**) at the same time, in the nucleus, 5S pre-RNA is transcribed by RNA polymerase III and then imported into the nucleolus. Riboproteins (RP) and other factors, including ribo-binding proteins (RBP) and eukaryotic translation initiation factor 6 (eIF6), are transcribed by RNA polymerase II and then translated into the cytoplasm, before being imported into the nucleolus. In the nucleolus, after the complete removal of ITS and ETS, 18S rRNA is taken charge of by several small ribosomal subunit proteins (RPS), including RPS14, which is mutated in 5q, and RPSA, which is mutated in isolated congenital asplenia (ICA) and RBPs, including EMG1 (mutated in Bowen–Conradi syndrome (BCS)) and UTP4 (mutated in North American Indian childhood cirrhosis (NAIC)), to produce the pre-40S ribosome subunit. In parallel, 28S, 5.8S, and 5S are taken charge of by several large ribosomal subunit proteins (RPL) and RBPs to produce the pre-60S ribosomal subunit. DBA, characterized by mutations in either RPLs or RPSs, impacts both pre-40S and 60S subunit biogenesis. Maturation of both pre-subunits continues through separate pathways first in the nucleolus, then in the nucleus to produce mature 40S and 60S subunits in the cytoplasm; (**D**) the 40S subunit, associated with several eIFs, scans mRNAs until encountering start codon AUG complementary to the Met-tRNA present in its P site. The 40S subunit’s eIFs are removed before the 60S subunit, and the last stage of maturation consists in eIF6 release by SBDS (mutated in Shwachman–Diamond syndrome (SDS)) complexes with the 40S subunit to produce the fully functional ribosome. Given the variety of mutations responsible for neurodevelopmental disorders (ND), proteins are involved in almost every stage in ribosome biogenesis.

Biosynthesis of this highly intricate ribonucleoprotein complex is challenging, given all its components. Two pre-RNAs, 47S and 5S, are transcribed and processed sequentially by several ribosomal and non-ribosomal proteins in the nucleolus to produce the pre-40S and 60S subunits. These pre-subunits are exported to the nucleoplasm, then to the cytoplasm, through separate maturation pathways involving multiple factors. Once matured in the cytoplasm, the newly-formed 40S and 60S subunits can combine during initiation of mRNA translation, to form the final 80S ribosome [3,4,5] (Figure 1).

Considering the importance of ribosomes, their formation is therefore a critical process for the cell. Impairment in ribosome biogenesis and/or function is associated with diseases called ribosomopathies. These genetic disorders are not only rare but also as varied as the genes impaired by the mutations. Despite the need for ribosomes in cells of any tissue, the clinical impacts observed in ribosomopathies are tissue-specific [6,7].

Since the discovery of the first ribosomopathy, X-linked dyskeratosis congenita, many others have been described, but only a few are solely responsible for an altered function of the ribosome. Some of the gene mutations implicated cause a lack of function called haploinsufficiency, resulting in biological disorders where expression of both alleles is needed. Breakthroughs in the sequencing field now make it possible to link ribosome involvement to other apparently causeless diseases (5q-syndrome, isolated congenital asplenia), and, thus, identify mutations causing the defective proteins integrated into ribosomes during biogenesis. Recently, we identified de novo mutations responsible for the expression of abnormal variants of the 60S subunit protein RPL13 (eL13). These proteins are efficiently integrated, giving “mutated” ribosomes responsible for severe bone defects [8].

Although these mutations do not alter fetal development, they are responsible for various severe phenotypes, including early lethality. Finding therapeutics to treat such pathologies is a real issue, not only because of the very disabling clinical disorders, but also the fact that ribosomopathies are known to be involved in oncogenesis [9,10]. Cancer development and progression have been linked to ribosome biogenesis dysregulation, affecting the expression of key factors involved in tumorigenesis. One of the major mechanisms is the activation of p53 through the MDM2-p53- riboproteins (RP) pathway [11,12]. Likewise, in X-linked dyskeratosis congenita, *DKC1* loss of function is known to disrupt p27 [13] and p53 [14] translation driving cancer development.

The aim of this manuscript is to review the current and new therapeutic perspectives of certain ribosomopathies.

## 2. The Main Mechanisms of Ribosomopathies

In 1998, X-linked dyskeratosis congenita (DC) was mapped and mutations in *DKC1* impacting rRNA processing were identified [15]. It was the first time that defects in ribosomes had been correlated with the onset of a disease. Later, other mutations in genes encoding for ribosomal proteins, or associated proteins, were linked to the development of several diseases characterized by distinct and wide-ranging clinical features.

As already reviewed [16,17], mutations can either affect ribosomal proteins, the component of the final ribosome, or other proteins involved in the different stages in ribosome assembly. In Treacher Collins syndrome (TCS), rDNA transcription is affected. 47S pre-rRNA processing is disrupted in Diamond–Blackfan anemia (DBA), cartilage hair hypoplasia (CHH) and X-linked dyskeratosis congenita (XL-DC). Pre-40S ribosomal subunit assembly and 18S rRNA processing are affected in Bowen–Conradi syndrome (BCS), North American Indian childhood cirrhosis (NAIC), isolated congenital asplenia (ICA), and 5q-syndrome (5q). In DBA, pre-40S and 60S ribosomal subunit assembly, including pre-rRNA processing, is disrupted. Final maturation of the 60S ribosomal subunit is affected in Shwachman–Diamond syndrome (SDS). Ribosomopathies causing neurodevelopmental disorders (ND) may be involved at each stage of ribosome biogenesis (Figure 1). Surprisingly, no pattern can be defined concerning the ribosome biogenesis stage affected and the tissues impacted in ribosomopathies. Ribosome biogenesis can be disturbed during pre-rRNA transcription and modification, pre-rRNA processing or ribosome assembly, while p53-mediated nucleolar stress has been associated with some manifestations of ribosomopathies [16].

Currently, three hypotheses co-exist to explain that these wide-ranging defects impair specific tissues [16]. The first is that the concentration of ribosomes could impact the specificity of mRNA translation [18]. The second is the “specialized ribosome” theory: a variable stoichiometry of ribosomal proteins within the ribosome would make possible the translation of some sub-pools of mRNA [19]. Finally, the last hypothesis is that changes in ribosome quantity or function are a secondary and compensatory effect of the expression of certain genes [20].

## 3. Ribosomopathies with Specific Corrections

### 3.1. Diamond–Blackfan Anemia (DBA)

Diamond–Blackfan anemia (OMIM #105650, https://omim.org/) is an inherited form of red blood cell aplasia occurring in the first year of life. The main clinical features of the disease are normochromic and macrocytic anemia, reticulocytopenia, and a near absence of erythroid progenitors in the bone marrow [21]. The disease is associated with growth retardation and, in 30 to 50% of cases, with congenital malformations (craniofacial, upper limb, heart, and urinary system) [22]. It has been associated with several heterozygous mutations in genes encoding for riboproteins of the 40S (RPS7-10-17-19-24-26) and 60S (RPL3-5-9-10-10A-11-15-18-19-26-34-35-35A and RPLP0) subunits [23,24,25,26,27,28,29,30,31,32,33]. The disease may also be secondary to copy number variations for some of the previously mentioned genes (*RPS17*-*19*-*24*-*26* and *RPL5*-*11*-*15*-*35A*) [30,34,35,36]. These mutations cause haploinsufficiency in the proteins, which leads to impairment in the synthesis of ribosomal subunits and pre-rRNA processing [26,27,29,37,38,39,40] (Figure 1). Mutations in *GATA1* (Gene ID: 2623, https://www.ncbi.nlm.nih.gov/gene), encoding for a key transcription factor in the erythroid lineage, have also been linked to the development of DBA [41]. Recently, it has been shown that *GATA1* mRNA, given the length and conformation of its 5′UTR, is down translated in the context of ribosome-reduced levels associated with *TSR2* (Gene ID: 90121) haploinsufficiency in DBA. Erythropoiesis is consequently altered [18] (Table 1). This was the first suggestion of the existence of a tissue-specificity pattern associated with ribosomopathy.

### 3.2. Current Treatment

Currently, patients are treated first with transfusions and then with corticosteroids [42]. Steroid-resistant and transfusion-dependent patients are then treated with hematopoietic stem cell transplantation (HSCT) [21], with a better outcome when the stem cells come from siblings [43] (Table 1).

Depending on the side effects from the steroid treatment (hypertension, diabetes mellitus, and growth retardation), and the heaviness of the immunosuppressive therapy associated with HSCT, alternatives have been investigated.

### 3.3. Therapeutic Perspectives

The amino acid L-leucine improves anemia and developmental defects in different DBA models (hCD34+, zebrafish, mouse), activating mRNA translation through modulation of the mechanistic target of the rapamycin (mTOR) pathway [44,45]. The mTOR has been shown to regulate the translation of TOP mRNAs characterized by a 5-terminal oligopyrimidine tract (TOP). These mRNAs encode for components of the translational apparatus particularly involved in initiating the translation [46].

Activin receptor type IIA ligand trappers such as Sotatercept (ACE-011), which are efficient on erythropoiesis in patients, were first developed for β-thalassemia patients [47]. RAP-011, the murine orthologue of Sotatercept, has been found to increase erythroid cells in zebrafish DBA by sequestering lefty1 (Lft1), an activin/nodal antagonist, upregulated in these models. Despite its incomplete description, Lft1 seems to delay maturation of erythroid cells [48]. As hypothesized, Sotatercept may increase erythropoiesis by blocking the inhibitory signals induced by members of the transforming growth factor beta (TGF-β) superfamily [49].

Trifluoperazine (TFP), a calmodulin inhibitor, induces an increase in hemoglobin levels in different DBA models (hCD34+, zebrafish). In a context of riboprotein deficiency, TFP seems to reduce the phosphorylation of p90 ribosomal S6 kinase (RSK) family members, which are hyper-phosphorylated in this case, and the downstream signaling pathway of these proteins leading to a reduction in p53 activity and rescue of the DBA phenotype [50].

Using induced pluripotent stem cells (iPSC) reprogrammed from DBA patients, drug screening has been carried out and SMER28, a small autophagy-promoting molecule, has been identified. SMER28 has been shown to act through ATG5, a factor required for autophagosome assembly, stimulate erythropoiesis, and up-regulate the expression of globin genes [51]. Nevertheless the associated mechanisms appear unclear because of the controversial autophagy status in DBA [52].

Once pancytopenia is reached in DBA, the only effective treatment is HSCT. In 2012, a DBA patient presenting progressive pancytopenia was treated with eltrombopag (EPAG), a thrombopoietin (TPO) receptor agonist, which seemed to increase both the hemoglobin rate and number of platelets [53]. It is known that TPO agonists can activate the JAK2/STAT5 pathway which results in an increase in megakaryocyte progenitor proliferation and platelet production [54].

Danazol, a synthetic androgen compound that inhibits pituitary gonadotrophins, had a positive effect on hemoglobin levels in DBA patients [55]. This follow-up is based on the observation that males have higher hemoglobin levels than females [56], although the mechanisms of action of Danazol remain unclear. This treatment, in association with corticosteroids, has already been used but was unfortunately responsible for the development of carcinomas in the patients treated [57].

Excluding Danazol, because of its side effects on growing children, all these compounds could be used to treat DBA (Table 1). L-leucine (NCT01362595, NCT02386267), Sotatercept (NCT01464164), TFP (NCT03966053), and EPAG (NCT04269889) (https://clinicaltrials.gov/ct2/home) are already being evaluated in clinical trials. It should be noted that all these treatments, except L-leucine, focus on bone marrow failures, which is just one of the many symptoms of DBA.

### 3.4. Dyskeratosis Congenita (DC)

Dyskeratosis congenita is an inherited disorder characterized by mucocutaneous abnormalities (skin pigmentation, nail dystrophy, and leucoplakia) and other features including, non-exhaustively, epiphora, developmental delay pulmonary disease, and short stature. Bone marrow failures occur in a very high proportion of DC cases, 93% of patients present peripheral cytopenia of at least one lineage [58]. There are three types of DC, depending on the genetic features: the autosomal dominant, characterized by mutations in *TERT* (Gene ID: 7015) [59,60] or *TERC* (Gene ID: 7012) [61], the X-linked form characterized by mutations in *DKC1* (Gene ID: 1736) [15], and the autosomal recessive form, characterized by mutations in genes encoding for the proteins associated with the telomerase complex including *GAR1* (Gene ID: 54433), *NHP2* (Gene ID: 55651) and *NOP10* (Gene ID: 55505) [62,63] (Table 1). While telomerase activity is responsible for the occurrence of the clinical features of autosomal forms of DC, ribosomal impairments are also described in X-linked DC (OMIM #305000) patients. It has been shown that dysfunctional dyskerin, a protein involved in pseudouridylation during rRNA processing [64,65] (Figure 1), disrupts the translation of mRNAs containing internal ribosome entry site (IRES) elements [66]. However, functional perturbations in XL-DC could be caused by telomere maintenance defects rather than rRNA pseudouridylation impairment [67].

### 3.5. Current Treatment

As in DBA, cytopenia is managed with chronic transfusions and, when patients become transfusion-dependent, HSCT is performed [63]. DC is highly pleiotropic, patients are monitored closely to screen for immunology, dermatology, neurology, ophthalmology, otolaryngology, dental, cardiology, and pulmonary complications [68] (Table 1).

### 3.6. Therapeutic Perspectives

Bone marrow failures are the principal targets of new therapeutic perspectives for DC. Currently, EPAG [69] and Danazol [70,71] are being investigated to improve the hematologic response in patients, as is the case in DBA. However, the effects of Danazol in DC are clearer than in DBA. It has been shown that androgens aromatized into estrogens can up-regulate *TERT* via nuclear hormone receptors leading to telomere elongation in patients with telomere diseases [72,73] (Table 1). Danazol has been tested during a clinical trial for DC and Fanconi anemia (NCT01001598. Only one DC patient was recruited but, despite the low number of patients, Danazol seemed to increase hemoglobin, platelets, and neutrophils.

### 3.7. Treacher Collins Syndrome (TCS)

Treacher Collins syndrome (OMIM #154500) is an autosomal dominant disorder affecting craniofacial development. It is characterized by gradual hearing loss, lateral downward sloping of the palpebral fissures, hypoplasia of the mandible, and presence of a cleft palate [74]. The disease arises during early embryonic development from abnormal neural crest cell formation and proliferation [75]. Several mutations in *TCOF1* (Gene ID: 6949), encoding for a nucleolar protein involved in ribosomal DNA gene transcription (Figure 1), have been identified [74]. Mutations cause premature stop codon appearance, leading to truncated forms of Treacle. As a result, Treacle is not correctly addressed in the cell, especially its recruitment into the nucleolus [76,77]. As a consequence, RNA polymerase I is not recruited to DNA, leading to failure in rDNA transcription [78]. Moreover, mutations in genes encoding subunits of RNA polymerase I and III, *POLR1C* (Gene ID: 9533) and *POLR1D* (Gene ID: 51082), have been associated with TCS features [79,80] enforcing the idea that TCS is caused by a disruption in rDNA transcription (Table 1).

### 3.8. Current Treatment

TCS is currently managed by treating the clinical manifestations in relation to the patient’s specific needs. A multidisciplinary craniofacial management team handles the different symptoms with reconstructive surgery and speech therapy [81] (Table 1).

### 3.9. Therapeutic Perspectives

To avoid surgical procedures, alternatives have been investigated. Recently, it has been demonstrated that proteasome inhibitors prescribed in multiple myeloma [82,83], such as MG132 and Bortezomib, were able to reduce craniofacial skeleton malformation in a zebrafish model of TCS, through a decrease in the degradation of the cellular nucleic acid-binding protein (Cnbp) [84]. Cnbp is required for proper craniofacial development and improves TCS craniofacial abnormalities by reducing the redox-responsive gene pathway, activated in TCS [85] (Table 1).

### 3.10. Cartilage Hair Hypoplasia (CHH)

Cartilage hair hypoplasia (OMIM #250250) is a pleiotropic autosomal recessive disorder. Defects include short stature, hypoplastic hair, ligamentous laxity, defective immunity, hypoplastic anemia, and neuronal dysplasia of the intestine. In less frequent cases, patients can also suffer from Hirschsprung’s disease (or congenital megacolon) [86]. The disease was first observed in the Amish community with an incidence of 1:1340, although no precise measure of the worldwide incidence has yet been made [87]. However, different carrier frequencies have been established and are up to 1:19 in Amish [87] and 1:76 in Finnish [88] communities. CHH is caused by various mutations in the RMRP gene (Gene ID: 6023) encoding for an RNA component of mitochondrial RNA processing endoribonuclease, essential for the cleavage of several RNA substrates during rRNA processing [89] (Table 1). These mutations lead to RMRP promoter inefficiency or RNA transcript instability [90] (Figure 1). The RMRP gene, whose promoter responds to chondrogenic morphogens, is therefore regulated during chondrocyte differentiation. Its impairment probably plays a role in the physiopathology of CHH [91]. RMRP mutations have been associated with inhibition of intramembranous ossification of skull bones and activation of vertebrae ossification. Moreover, disrupted RMRP inhibits cell proliferation, promotes apoptosis and activates the Wnt/β-catenin pathway, probably by suppressing the degradation of β-catenin [92].

### 3.11. Current Treatment

Neutropenia is managed using injections of granulocyte colony stimulating factor (G-CSF) [93], while immunodeficiency is corrected by bone marrow transplant [94] (Table 1). These treatments do not take into consideration the other aspect of CHH: chondrodysplasia.

### 3.12. Therapeutic Perspectives

In 2013, recombinant growth hormone injections made it possible to increase one CHH patient’s height to near age-normal [95], while growth hormone treatment has already been associated with improved growth and immune systems in patients [96]. Although promising in CHH management, using this type of hormonal therapy appears risky because of the probable side effects. Moreover, to date, only one case has been reported and the results need to be confirmed with a larger number of subjects to assess not only the efficiency, but also the safety, of using recombinant growth hormone in CHH patients.

XAV939, an inhibitor of Wnt/β-catenin signaling through stimulation of β-catenin degradation, via GSK3/Axin complex stabilization, seemed to partially alleviate chondrodysplasia and increase vertebrae mineralization in zebrafish RMRP mutants [91] (Table 1).

### 3.13. Shwachman–Diamond Syndrome (SDS)

Shwachman–Diamond syndrome (OMIM #260400) is a pleiotropic autosomal recessive disorder. Patients suffer from various hematological (neutropenia, anemia, thrombocytopenia, bone marrow hypoplasia) and non-hematological (skeletal abnormalities, pancreatic defects) symptoms [97]. In 90% of cases, mutations in *SBDS* (Gene ID: 51119), encoding for a protein involved in ribosome biogenesis, are responsible for SDS [98] (Figure 1). SBDS, associated with EFL1, directly catalyzes eukaryotic initiation factor 6 (eIF6) removal, which is necessary for binding 40S and 60S ribosomal subunits to produce functional 80S ribosomes. Loss of SBDS expression leads to eIF6 retention, causing impairment to 80S ribosome assembly [99,100]. Recently, other mutations in *DNAJC21* (Gene ID: 134218) [101], *EFL1* (Gene ID: 79631) [102] and *SRP54* (Gene ID: 6729) [103] have been associated with SDS (Table 1). As reviewed by [104], these mutations also alter eIF6 release.

### 3.14. Current Treatment

SDS is currently treated on the basis of consensus guidelines. Cytopenia is managed with chronic transfusions associated with an iron chelation program. In cases of severe neutropenia, G-CSF is used to prevent infections. HSCT is only considered when patients do not respond to G-CSF. Because of its highly pleiotropic pattern, SDS is managed with several medical acts [105] (Table 1).

### 3.15. Therapeutic Perspectives

Recently, TGF-β signaling has been found to be activated in SDS stem and multipotent progenitors associated with suppression of hematopoiesis [106]. Its inhibition with different compounds, AVID200 and SD208, increased hematopoietic colony formation in SDS bone marrow cells [106]. TGF-β signaling is in fact suppressed during erythropoiesis, explaining why inhibitors of this signaling pathway are evaluated to improve anemia in myelodysplastic syndromes [49].

In 50% of cases, mutation c. (183–184 TA > CT) of *SBDS* causes premature termination codon (PTC) in SDS patients [98]. Ataluren, a compound that allows the ribosome to go past the PTC, has been shown to restore SBDS expression in patients’ myeloid cells. Moreover, Ataluren therapy has been associated with an increase in SBDS protein expression, myeloid precursor differentiation and a decrease in the apoptotic rate of peripheral blood mononuclear cells PBMCs from patients [104] (Table 1).

### 3.16. 5q-Syndrome (5q)

5q-syndrome (OMIM #153550) is a deletion of the q (q31 and q33) arm of chromosome 5 in cancer. Symptoms are macrocytic anemia, erythroid hypoplasia in the bone marrow, and hypolobated micromegakaryocytes. Some patients also evidence elevated platelet and reduced neutrophil counts. 5q is caused by a loss of function in the RPS14 component of the 40S ribosomal subunit (Table 1), which abrogates 40S subunit formation by blocking 30S pre-rRNA processing [107] (Figure 1).

### 3.17. Current Treatment

The 5q is managed therapeutically with red blood cell transfusions replaced progressively by recombinant erythropoietin, thalidomide, and retinoid injections. Chemotherapy, hypomethylating agent injections, and allogenic bone marrow transplants, are also part of the treatment [108]. Currently, lenalidomide, derived from thalidomide, is used as a gold standard to treat transfusion-dependent 5q patients [109] (Table 1). Lenalidomide promotes p53 degradation, which is over-activated in response to nucleolar stress in the context of the disease, by inhibiting the auto-ubiquitination of MDM2 (Gene ID: 4193) through decreased expression of *PPP2Acα* (Gene ID: 5515), encoding for the protein phosphatase 2 catalytic subunit alpha. Thus, the *PPP2Acα* over-expression found in some patients promotes lenalidomide drug resistance though restored p53 activation [110]. Given this resistance in certain patients, alternative treatments are being investigated.

### 3.18. Therapeutic Perspectives

Cenersen, a 20-mer antisense oligonucleotide, has been evaluated in patients with hematological malignancies, including acute myeloid leukemia and chronic lymphocytic leukemia [111,112,113]. Cenersen binds to *p53* mRNA which undergoes subsequent cleavage by ribonuclease H [114]. This antisense oligonucleotide reduces p53 and the p53-up-regulated modulator of apoptosis in *RPS14*-deficient erythroblasts, resulting in improved cell growth. Although the suppression of the 5q clone has not been observed, Cenersen treatment promotes erythroid colony-forming capacity [115]. Cenersen is currently being investigated in a clinical trial (NCT02243124).

As in DBA, L-leucine has also been shown to improve anemia in 5q [44] (Table 1).

## 4. Ribosomopathies Treated with Generic Guidelines

### 4.1. North American Indian Childhood Cirrhosis (NAIC)

North American Indian childhood cirrhosis (OMIM #604901) is an autosomal recessive disease. Newborn patients suffer only from transient jaundice, which progresses to biliary cirrhosis and portal hypertension [116]. Although no cause for NAIC has yet been found, biochemical, and histopathological features of the disease suggest that the bile ducts are involved. Mutations in the *UTP4* gene (Gene ID: 84916), more frequently called *NAIC* or *CIRH1A*, are responsible for the disease [117]. This gene encodes for a WD40 repeat-containing protein in the small subunit processome involved in the maturation of the ribosome’s 18S rRNA [118] (Figure 1). Currently, the only effective treatment for NAIC is a liver transplant [117] (Table 1).

### 4.2. Isolated Congenital Asplenia (ICA)

Isolated congenital asplenia (OMIM #271400) is an autosomal dominant disease characterized by an absence of spleen at birth. Given the role of the spleen in the immune system, this major developmental defect makes patients prone to life-threatening bacterial infections [119]. ICA is caused by mutations in *RPSA* (Gene ID: 3921), encoding for a protein in a small subunit of the ribosome [120] (Figure 1). Current guidelines for treating infections in patients with asplenia, including ICA, consist of anti-infection and antibiotic prophylaxis but also multiple vaccinations (pneumococcal, *Haemophilus influenzae* type b, meningococcal, and influenza) [121] (Table 1).

### 4.3. Neurodevelopmental Disorders (ND)

Neurodevelopmental disorders are a wide spectrum of diseases in which structural and/or functional development of the nervous system is disrupted, and/or neurodegeneration occurs in childhood. These disorders cover various phenotypes, including brain malformations such as neural tube defects or microcephaly, autism spectrum disorders, schizophrenia, epilepsy, and cerebral palsy/periventricular leukomalacia [122,123]. Given the consequences of ribosome biogenesis dysregulation in nervous system development, as reviewed by [124], several mutations in genes encoding for riboproteins or proteins involved in ribosome biogenesis have been identified as causing ND. Mutations in *RPL10* (Gene ID: 6134) have been associated with the onset of autism [125], intellectual disability associated with cerebellar hypoplasia and spondyloepimetaphyseal dysplasia [126], microcephaly [127], and mutations in *RPS23* (Gene ID: 6228) associated with microcephaly and hearing loss combined with growth deficits and dysmorphic features [128]. Other mutations causing ND affect *LAS1L* (Gene ID: 81887) [129], *SMN1* (Gene ID: 6606) [130], *EXOSC3* (Gene ID: 51010) [131] and *UBTF* (Gene ID: 7343) [132,133]. All of these ribosomopathies have been reviewed by [124]. Depending on the manifestations and difficulties affecting patients, their mental health is monitored closely, including medication, but they can also receive speech therapy, as well as pediatric and educational help [123] (Table 1).

## 5. Ribosomopathies that Remain Untreated

### Bowen-Conradi Syndrome (BCS)

Bowen-Conradi syndrome (OMIM #211180) is an autosomal recessive developmental disorder. Patients present pre- and postnatal psychomotor defects, growth retardation, microcephaly, micrognathia, and congenital vertical talus. BCS leads to early death [134]. A single amino-acid exchange (aspartate to glycine) in position 86 of EMG1 is responsible for causing BCS. This nucleolar RNA methyltransferase is involved in the biogenesis of a small subunit of the ribosome, and its mutation causes an absence from its pre-ribosomal binding sites in the nucleus. EMG1 is no longer present at its active site, which blocks 18S rRNA processing (Figure 1). Given the mortality of the disease, there is currently no treatment for managing the symptoms [135] (Table 1).

## 6. Conclusions

Ribosomopathy is a term that covers a wide range of pathologies. They can be caused by a multiplicity of mutations that remain specific to one disease. Currently, most of these pathologies are managed on the basis of generic therapeutic guidelines. However, there is still a need to search for new treatments for ribosomopathies to avoid the side effects associated with existing drugs or the heaviness of certain therapies (multiplicity/chronicity of procedures, surgery, and transplants). This need is also due to secondary diseases, such as cancer, which can arise from ribosomopathies.

**Figure 2:**
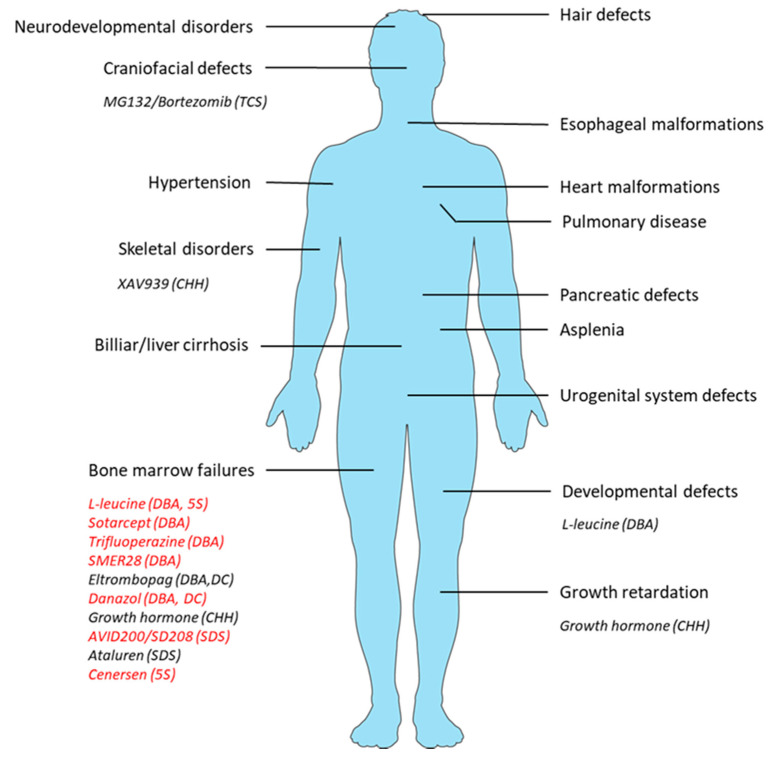
Clinical features of the main ribosomopathies and targets of the new therapeutic perspectives. Therapies that only improve erythropoiesis are indicated in red.

The physiopathological diversity of ribosomopathies described in this manuscript show the difficulty in designing appropriate therapies for handling this group of rare genetic disorders. Ribosomopathies are also characterized by their clinical features, affecting specific tissues, such as the bone marrow compartment in DBA. Drugs, surgical acts, and psychological therapy for managing the symptoms of ribosomopathies are thus borrowed from diseases, which encompass common clinical aspects. This is even true for the new therapeutic perspectives (Figure 2).

However, recent progress in gene and cell therapy will make it possible to treat clinical disorders and re-establish normal ribosome function in tissues that are easy to manipulate. Thus, in the bone marrow compartment, it has been shown that gene delivery by retrovirus and lentivirus can correct the phenotype in RPS19-deficient human cells [136], as well as in preclinical mouse models [137,138]. More recently, phenotypes caused by RP mutations were rescued by gene therapy in lymphoblastoid cell lines (LCLs) established from RPS19-deficient DBA patients. Protein synthesis increased and levels of p53 dropped in the LCLs [139]. Thus, in numerous ribosomopathies, where bone marrow failure syndromes are a common feature, they could be managed using gene therapy. Recent progress in genome editing (Zinc finger nucleases (ZFNs), Transcription activator-like effector nuclease (TALEN), and CRISPR/Cas9) is also promising in the treatment of the clinical disorders in ribosomopathies. CRISPR/Cas9 technology has been used to target cells from various tissues, such as hematopoietic stem cells [140], skeletal muscle cells [141], or inner ear cells [142]. We can speculate that in the next few years, new strategies using gene editing to correct the disorders found in ribosomopathies will appear by correcting mutations in specific tissues. RNA-based strategies are also an alternative to genome editing and might be developed in DBA in the future [143], although these strategies require early diagnosis and medical care, in order to avoid the multiplication of clinical disorders.

In the meantime, a significant number of ribosomopathies are the result of de novo mutations, which means that they cannot be predicted before fertilization. Despite this, genetic counseling can be provided after birth to identify the mutation, delineate the resulting disease, and introduce the appropriate treatment. Identifying cures for these diseases will be a challenge. Moreover, understanding the mechanisms of ribosomopathies will make it possible to find suitable therapeutic targets and manage diseases differently, thus improving the lives of patients. Explaining the tissue-specificity of ribosomopathies is also challenging and will expand the possibilities of proposing target therapy associated with specific drug delivery to the affected tissue. In the past few years, studies on ribosomopathies have refocused on the crucial role played by ribosome function in cell biology [144]. Given the large number of diseases with translational defects [145], fundamental research on this key cellular function appears necessary.

## Figures and Tables

**Table 1:** Ribosomopathies, main mutated genes, main clinical features, current treatment and therapeutic perspectives.

Name	OMIM	Mutations		Phenotype	Current Treatment	Therapeutic Perspectives
Diamond–Blackfan anemia	105650	•*RPS7*	•*RPL10*A	•Anemia	•Chronic transfusions	•Danazol
		•*RPS10*	•*RPL11*	•Growth retardation	•Steroids	•L-leucine
		•*RPS17*	•*RPL15*	•Other defects ~30–50%	or	•Sotatercept
		•*RPS19*	•*RPL18*	•Craniofacial	• Hematopoietic Stem Cell Transplantation (HSCT)	•Trifluoperazine
		•*RPS24*	•*RPL19*	•Upper limb		•SMER28
		•*RPS26*	•*RPL26*	•Heart malformations		•Eltrombopag
		•*RPLP0*	•*RPL34*	•Urinary system malformations		
		*•RPL3*	•*RPL35*			
		•*RPL5*	•*RPL35A*			
		•*RPL9*	•*TSR2*			
		•*RPL10*				
X-linked dyskeratosis congenita	305000	*•DKC1*		•Skin pigmentation	•Chronic transfusions	•Eltrombopag
				•Nail dystrophy	•HSCT	•Danazol
				•Leucoplakia	•Regular examinations in	
				•Cytopenia	•Immunology	
				•Other defect >30%	•Dermatology	
				•Epiphora	•Neurology	
				•Learning difficulties/mental retardation	•Ophthalmology	
				•Pulmonary disease	•Otolaryngology	
				•Hyperhidrosis	•Dental	
				•Extensive dental carries/loss	•Cardiology	
				•Short stature	•Pulmonary	
				•Hair loss/grey hair or sparse eyelashes		
				•Esophageal stricture		
				•Hypogonadism/undescended testes		
				•Urethral stricture/phimosis		
				•Malignancy		
				•Liver cirrhosis/adenoma		
				•Abnormal bone trabeculation/osteoporosis		
Treacher Collins syndrome	154500			•Defects of craniofacial development	•Reconstructive surgery	•MG132 or Bortezomib
		•*TCOF1*		•Conductive hearing loss	•Speech therapy	
		•*POLR1C*		•Palpebral fissures’ lateral downward sloping		
		•*POLR1D*		•Mandible hypoplasia		
				•Cleft palate		
Cartilage hair hypoplasia	250250	•*RMRP*		•Short stature	•Granulocyte Colony-Stimulating Factor (GCSF)	•Recombinant growth hormone
				•Hypoplastic hair	•HSCT	•XAV939
				•Ligamentous laxity		
				•Defective immunity		
				•Hypoplastic anemia		
				•Neuronal dysplasia of the intestine		
Shwachman–Diamond syndrome	260400			•Neutropenia	•Chronic transfusions	•Transforming Growth Factor beta (TGF-β)
		*•SBDS*		•Anemia	•Androgens	•Ataluren
		*•DNAJC21*		•Thrombocytopenia	•HSCT	
		*•EFL1*		•Bone marrow hypoplasia	•Reconstructive surgery	
		*•SRP54*		•Skeletal abnormalities	•Pancreatic enzymes	
				•Pancreatic defects	•Vitamin supplements	
					•Dietary advice and surveillance	
5q-syndrome	153550	•*RPS14*		•Macrocytic anemia	•Red blood cell transfusions	•Cenersen
				•Erythroid hypoplasia	•Recombinant erythropoietin	•L-leucine
				•Hypolobated micromegakaryocytes	•Thalidomide	
					•Retinoids	
					•Chemotherapy	
					•Hypomethylating agents	
					•Bone marrow transplantation	
North American Indian	604901	•*UTP4*		•Natal transient jaundice	•Liver transplantation	
childhood cirrhosis				•Biliary cirrhosis		
				•Portal hypertension		
Isolated congenital asplenia	271400	•*RPSA*		•Absence of spleen	•Anti-infection and antibiotic prophylaxis	
					•Vaccination	
					•Pneumococcal	
					•*Haemophilus influenzae* type b	
					•*Meningococcal*	
					•*Influenza*	
Neurodevelopmental disorders		*•RPL10*	*•POLR1A*	•Neural tube defects	•Mental health medication	
		*•RPS3*	*•ERCC6*	•Microcephaly	•Speech therapy	
		*•LAS1L*	*•CSB*	•Autism	•Pediatrics	
		*•SMN1*	*•ERCC8*	•Schizophrenia	•Educational help	
		*•EXOSC3*	*•CSA*	•Epilepsy		
		*•UBTF*		•Cerebral palsy/periventricular leukomalacia		
Bowen–Conradi syndrome	211180	•*EMG1*		•Psychomotor defects		
				•Growth retardation		
				•Microcephaly		
				•Micrognathia		
				•Congenital vertical talus		
